# Alanine/EPR dosimetry for mailed intercomparison at ocular proton therapy facilities—preliminary results for three centres for irradaitions at CCB IFJ PAN eyeline

**DOI:** 10.1093/rpd/ncad127

**Published:** 2023-09-18

**Authors:** Barbara Michalec, Cinzia De Angelis, Gabriela Foltyńska, Tomasz Horwacik, Brigitte Reniers, Agnieszka Wochnik, Renata Kopeć, Jan Swakoń

**Affiliations:** Institute of Nuclear Physics Polish Academy of Sciences, Radzikowskiego 152, 31-342 Krakow, Poland; Istituto Superiore di Sanità, Viale Regina Elena 299, 00161 Rome, Italy; Institute of Nuclear Physics Polish Academy of Sciences, Radzikowskiego 152, 31-342 Krakow, Poland; Institute of Nuclear Physics Polish Academy of Sciences, Radzikowskiego 152, 31-342 Krakow, Poland; Research Group NuTeC, Hasselt University, Agoralaan Gebouw H, B-3590 Diepenbeek, Belgium; Institute of Nuclear Physics Polish Academy of Sciences, Radzikowskiego 152, 31-342 Krakow, Poland; Institute of Nuclear Physics Polish Academy of Sciences, Radzikowskiego 152, 31-342 Krakow, Poland; Institute of Nuclear Physics Polish Academy of Sciences, Radzikowskiego 152, 31-342 Krakow, Poland

## Abstract

Quality control of therapeutic photon beams in the form of postal dose audits based on passive dosemeters is widely used in photon radiotherapy. On the other hand, no standardised dosimetry audit programme for proton centres has been established in Europe so far. We evaluated alanine/EPR dosimetry systems developed at the Istituto Superiore di Sanità (Italy), the Hasselt Universiteit (Belgium) and the Henryk Niewodniczanski Institute of Nuclear Physics Polish Academy of Sciences (Poland) for their applicability as a potential tool for routine mailed dose audits of passively scattered therapeutic proton beams. The evaluation was carried out in the form of an intercomparison. Dosemeters were irradiated in the 70 MeV proton beam at ocular proton therapy facility in the Cyclotron Centre Bronowice at the Henryk Niewodniczanski Institute of Nuclear Physics Polish Academy of Sciences in Krakow. A very good agreement was found between the dose measured by three laboratories and the delivered dose determined with an ionisation chamber. This, together with the inherent properties of alanine, such as non-destructive readout, tissue equivalence, weak energy dependence, dose rate independence and insignificant fading, makes alanine a good candidate for a dosemeter used in postal auditing in proton ocular radiotherapy.

## Introduction

Independent, external evaluations of radiotherapy procedures, in particular dose delivery procedures, are one of the key elements in quality assurance (QA) in radiation oncology. Their goal is both to control and improve the quality of radiotherapy process. Quality control (QC) and QA programmes and standards for high-energy protons and electrons are widely used and well recognised^(1)^. Independent dosimetry audits in photon therapy are nowadays a part of the clinical routine^(2)^. Inter-institutional dose delivery evaluation and proofing of the integrity of therapeutic dose delivery are also often required by national regulations^(3)^. On the other hand, in Europe, there are no standardised external QA and QC procedures in proton radiotherapy, although this therapeutic modality has been increasingly used for more than 20 y^(4)^. Currently, each of the operating proton radiotherapy facilities, usually in cooperation with other centres, has developed its own system of both internal and external QC to ensure the accuracy of its therapeutic procedures, including dose delivery^(5)^. So far, many inter-institutional dosimetry comparisons, based on active or passive dosemeters, have also been made, mainly for scanning proton beams^(6, 7)^. None of the methods used in these measurements has been formally indicated and recommended for external evaluation of therapeutic proton beams. However, many of them point to alanine as a good and promising candidate for a dosemeter routinely used in external comparisons and audits, also in a convenient postal form^(8, 9)^.

In view of developing a dose delivery evaluation programme for ocular proton therapy facilities, using mainly passively scattered beams, an exercise with alanine dosemeters at the eyeline in the Cyclotron Centre Bronowice at the Henryk Niewodniczanski Institute of Nuclear Physics Polish Academy of Sciences in Krakow (CCB IFJ PAN) has been performed.

**Table 1:** Alanine/EPR dosimetry systems used in the intercomparison.

Institution	Dosemeters	Spectrometer	Read-out method	Calibration^a^
ISS	Gamma Service ϕ = 4.8 mm h = 3 mm m = 65 mg 96% alanine	Bruker ELEXSYS	Spectrum registered for a stack of three alanine pellets Three repeated measurements with a random order of pellets in a stack	Co-60 (Dw) Proton (Dw)
HU	Harwell ϕ = 4.8 mm h = 2.7 mm m = 59.8 mg 90.1% alanine	Bruker EMXmicro	Five separate spectra for each alanine pellet after subsequent equal rotation steps	Co-60 (D_w_) Proton (D_w_)
IFJ PAN	Synergy Health^b^ ϕ = 4.8 mm h = 3 mm m = 65 mg 96% alanine	Bruker ESP 300E	Five accumulated spectra for each alanine pellet at one position in the cavity Five measurements after manual rotation of ca. 70^o^	Proton (D_w_)

^a^Calibration in terms dose-to-water^(10)^.

^b^Former name of manufacturer was Gamma Service.

This paper describes the results of this exercise, which was carried out in the form of mailed dosimetry comparison amongst independent alanine/EPR dosimetry systems developed in three European laboratories, with the aim of evaluating the differences in the determination of the absorbed dose to water^(10)^ in passively scattered proton beams following both the individual laboratory procedures and two different approaches to proton dose calculation: by the correction for the radiation quality (k_Qproton_ factor for proton beam) or by the direct calibration in a proton beam. The exercise also included the designing, manufacturing and distribution of phantoms as well as the transport and shipment of non-irradiated and irradiated alanine pellets. It therefore constituted a final test (dry run) prior to carrying out a future international dosimetry intercomparison for ocular proton radiotherapy facilities.

## Materials and methods

### Alanine/EPR dosimetry systems

Alanine/EPR dosimetry uses the phenomenon of stable free radical generation in alanine exposed to ionising radiation. Their concentration can be evaluated by Electron Paramagnetic Resonance (EPR) spectrometry and is proportional to the absorbed dose.

Three institutions participated with their alanine/EPR systems in the reported intercomparison: the Istituto Superiore di Sanità (ISS, Italy), the Hasselt Universiteit (HU, Belgium) and the Henryk Niewodniczanski Institute of Nuclear Physics Polish Academy of Sciences (IFJ PAN). A brief characteristic of the above-mentioned systems is presented in Table 1. A more detailed description of system properties and measurement protocols used in each institution is given by De Saint-Hubert *et al*.^(8)^.

Two different approaches were used to determine the absorbed dose to water in the proton beam. ISS and IFJ PAN calibrated their alanine dosemeters directly in the proton beam. On the other hand, HU used Co-60 calibration and applied the correction for the radiation quality (k_Qproton_) to convert the results to proton dose^(8)^. Both proton calibration curves and k_Qproton_ determination were performed at CCB IFJ PAN eyeline, which is designated as the reference centre in the future international intercomparison for ocular proton therapy facilities.

### Preparatory work for the intercomparison

**Figure 1:**
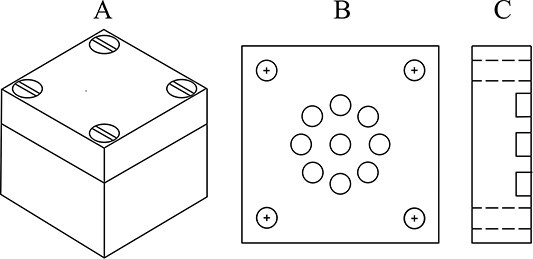
The scheme of the PMMA phantom used in the intercomparison for alanine irradiation. A - PMMA phantom; cover + base, B - cover bottom view, C - cover side view.

Dedicated, cubic-shaped, PMMA phantoms (Figure 1) were designed, manufactured and provided for each institution participating in the intercomparison. The phantom consisted of two parts: a cover (40 × 40 × 12 mm) and a base (40 × 40 × 28 mm). Nine cylindrical holes were drilled in the bottom part of the cover to house alanine pellets. Their size corresponded to the size of the pellets. The holes were placed in the centre of the cover, forming a circular area of 8 mm radius: one hole in its centre and the remaining eight symmetrically around it. This ensured a uniform distribution of dosemeters in a 25 mm homogeneous radiation field, for which a QC of the ocular facility is performed prior to clinical work. After loading the dosemeters, the cover and the base could be tightly connected with polyethylene screws.

Each laboratory participating in the intercomparison was provided with the phantom for individual assembly. After installing alanine pellets the phantoms were sent to IFJ PAN, where the reference irradiation was performed.

### Irradiation at CCB IFJ PAN ocular proton radiotherapy facility

**Figure 2:**
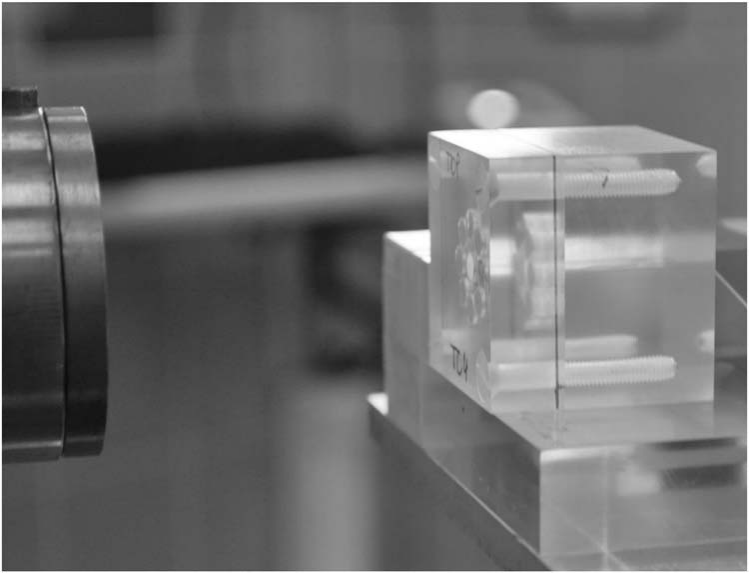
Intercomparison irradiation of the phantom with alanine dosemeters.

The intercomparison irradiation was performed at CCB IFJ PAN, at the clinically operating facility, where patients with ocular tumours routinely undergo their proton radiotherapy (Figure 2). The facility uses the passively scattered, horizontal proton beam produced by the Proteus C-235 isochronous cyclotron made by Ion Beam Application S.A. The initial proton energy of 230 MeV is degraded to 70 MeV and delivered to the treatment room, where the beam is formed according to clinical requirements.

Prior to the main irradiations, a determination of dose-to-water (calibration) was performed. The calibration was performed in the water phantom with the Semiflex ionisation chamber TM31010 (PTW Freiburg) connected to the reference class electrometer Unidos Webline (PTW Freiburg), according to IAEA TRS 398^(10)^.

The phantoms loaded with alanine pellets were then sequentially irradiated with a dose of 15 Gy in a homogeneous proton beam with a diameter of 25 mm, in fully modulated Spread Out Bragg Peak, at the depth of 13 mm water equivalent. During irradiation, dosemeters inside the phantoms were placed at the facility isocentre plane.

## Results

The phantoms with irradiated alanine dosemeters were sent back to the institutions participating in the intercomparison, where the pellets were taken out and processed according to the procedures established by each laboratory. As reported in Table 1 and in the paper by De Saint-Hubert *et al*.^(8)^, HU and IFJ PAN read out each of the nine dosemeters placed in the phantom individually, whereas ISS grouped the dosemeters into three stacks of three pellets and registered the EPR spectra from each stack^(11)^. Then each institution used its calibration (proton calibration curve or Co-60 calibration curve + k_Qproton_, as described in the previous section) to evaluate the absorbed dose from the EPR signal.

The dose value reported by HU and IFJ PAN was the mean of nine individual dose measurements registered by every single dosemeter in the phantom. In the case of ISS, it was the mean of three doses measured for each stack. The reported dose was finally compared with the reference dose measured with the ionisation chamber.

**Figure 3:**
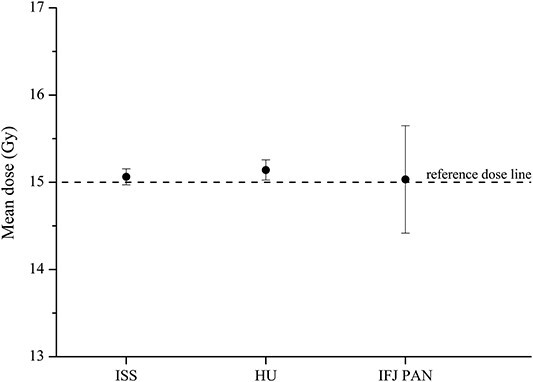
The mean alanine dose reported in the intercomparison by ISS, HU and IFJ PAN.

The dose values reported by each institution are shown in Figure 3 and Table 2. In Table 2, the dose delivered to each phantom with alanine pellet set is also reported.

**Table 2:** The mean dose measured by alanine dosemeters from ISS, HU and IFJ PAN versus the dose delivered to each irradiated phantom.

Institution	Mean alanine dose (Gy)	Delivered dose (Gy)	Difference between delivered and reported dose (%)
ISS	15.06 ± 0.10	15.00 ± 0.08	0.40
HU	15.14 ± 0.12	14.98 ± 0.08	1.07
IFJ PAN	15.03 ± 0.62	14.99 ± 0.08	0.27

## Discussion

Each of the three institutions involved in the intercomparison has developed its own alanine/EPR dosimetry system. These systems differ in the used dosemeters, EPR spectrometers, measurement methodology and the method of converting an EPR signal of alanine exposed to protons to the absorbed dose to water (Table 1). The dose value reported by the laboratories as an intercomparison result was calculated as the mean of individual dose measurements registered by single alanine pellets (HU, IFJ PAN) or by a group of dosemeters (ISS), depending on the procedure developed by each institution. As shown in Figure 3 and Table 2, the agreement between the dose measured by the three institutions is <1%. The difference between the alanine dose and the delivered dose does not exceed 1.1%. A much higher level of uncertainty in dose assessment for IFJ PAN can be explained by the spectrometer instability (about 3.5%), which was described by De Saint-Hubert *et al*.^(8)^.

A very good agreement between the delivered and the reported dose was expected since both the alanine/EPR system calibration in protons and the reported comparison irradiation were performed at the same facility. The high level of agreement (achieved under specific conditions, including common route of traceability) confirms that the investigated alanine/EPR systems are a suitable tool to diagnose potential differences between the planned and delivered dose in the future intercomparison.

The objective of the exercise, which was to test the entire intercomparison process: from the manufacturing of the phantoms, alanine pellet transport, shipment, irradiation, return to the lab and read-outs, was successfully achieved.

## Conclusions

In the current study, three independent alanine/EPR dosimetry systems were evaluated for their use as a potential tool for routine mailed dose intercomparison. The results show that they are well prepared for the evaluation of dose delivery for passively scattered therapeutic proton beams and can be effectively used in future projects and QA programmes in proton therapy centres.

## Data availability statement

Data are available through the request of the corresponding author.

## Conflicts of interest

The authors declare no conflict of interest.
